# Neutrophils and Granulocytic MDSC: The Janus God of Cancer Immunotherapy

**DOI:** 10.3390/vaccines4030031

**Published:** 2016-09-09

**Authors:** Serena Zilio, Paolo Serafini

**Affiliations:** Department of Microbiology Immunology, University of Miami, Miami, FL 33146, USA; s.zilio1@med.miami.edu

**Keywords:** gMDSC, neutrophils, cancer, immunotherapy, sex

## Abstract

Neutrophils are the most abundant circulating blood cell type in humans, and are the first white blood cells recruited at the inflammation site where they orchestrate the initial immune response. Although their presence at the tumor site was recognized in the 1970s, until recently these cells have been neglected and considered to play just a neutral role in tumor progression. Indeed, in recent years neutrophils have been recognized to play a dual role in tumor development by either assisting the growth, angiogenesis, invasion, and metastasis or by exerting tumoricidal action directly via the secretion of antitumoral compounds, or indirectly via the orchestration of antitumor immunity. Understanding the biology of these cells and influencing their polarization in the tumor micro- and macro-environment may be the key for the development of new therapeutic strategies, which may finally hold the promise of an effective immunotherapy for cancer.

## 1. Introduction

**Figure 1:**
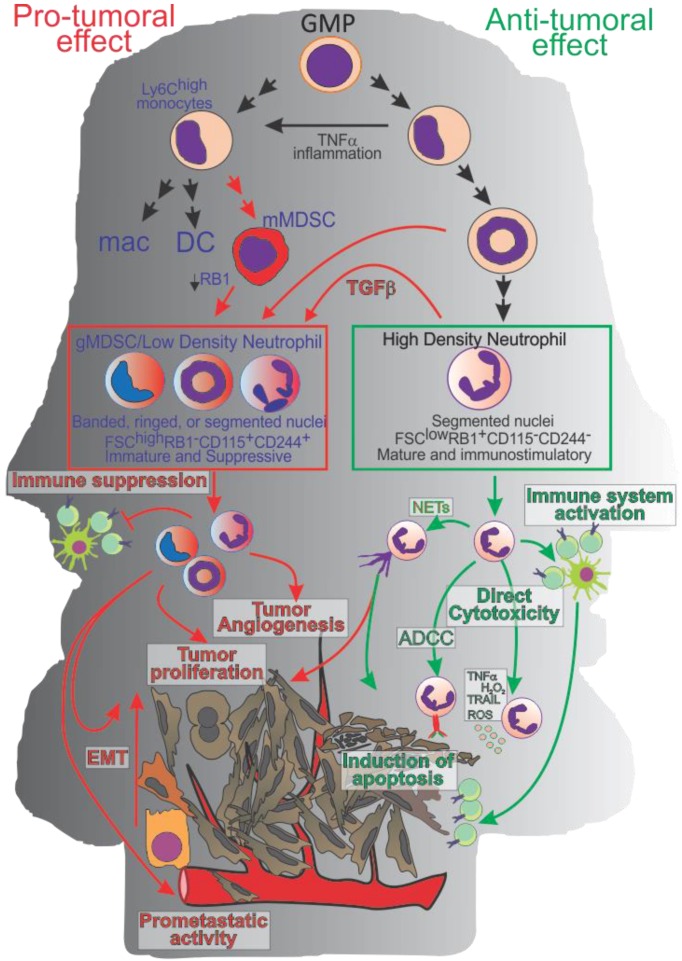
Neutrophil polarization and activity. In physiological conditions or during early stage of tumor progression Granulocyte-Macrophage Progenitor (GMP) cells commit toward mature High Density Neutrophils (HDN) able to exert an anti-tumor activity directly (via ROS, Reactive Nitrogen Species, Tumor Necrosis Factor-α TNF-α; TNF-Related Apoptosis-Inducing Ligand; TRAIL, anti-microbial protein or antibody-dependent cell-mediated cytotoxicity, ADCC) or indirectly by modulating the adaptive immune response. During cancer progression, Tumor Derived Factors (TDFs) modulate neutrophils and myeloid cell polarization toward a pro-tumoral phenotype (gMDSC and or low density neutrophils) characterized by an immature phenotype, by the capacity to inhibit the adaptive immune response, promote tumor angiogenesis, endothelial to mesenchymal transition (EMT), and metastases.

Initially described by Ehrlich in 1879 [1], and more clearly and better defined by Metchnikoff in 1893 as polymorphonuclear cells (PMN) possessing phagocytic activity and anti-microbial properties [2], neutrophils are still puzzling the scientific community today. Neutrophils are the most abundant white blood cells in the blood; because of their short lifespan (less than 24 h in physiological condition [3]), they are continuously generated in the bone marrow from myeloid precursors. Under normal conditions, granulocytes-monocytes progenitors (GMPs) can generate myeloblasts that pass through the phases of promyelocytes, myelocytes, ringed nuclei metamyelocyte and band cells to give rise to mature neutrophils [4]. However, under stress and inflammatory conditions, “normal” differentiation can be altered and, for example band cells [5] can transdifferentiate into monocytes and even develop into dendritic cells and macrophages (Figure 1). Conversely, suppressive monocytic like-cells (i.e., monocytic Myeloid Derived Suppressor Cells (mMDSCs)) can convert into suppressive neutrophil-like cells (or granulocytic Myeloid Derived Suppressor Cells (gMDSCs)) by an epigenetic mechanism that requires the downregulation of retinoblastoma (RB1) [6].

Throughout time, neutrophils have been considered the “kamikaze” cells that arrive first at the site of injury and immolate themselves while killing the invading pathogens with a variety of mechanisms that include phagocytosis, NETosis (active extrusion of DNA to generate Neutrophils Extracellular Traps (NETs)), secretion of reactive oxygen species (ROS), hyperchlorous acid, and antimicrobial proteins (i.e., defensin, lysozyme, and proteases such as elastase and cathepsin) [7]. However, a growing body of evidence is challenging this view, suggesting that neutrophils may exert a more complex role interacting with other components of the innate and adaptive immune system [8]. Indeed, neutrophils are associated with the pathogenesis of different inflammatory and auto-immune disorders [9,10,11,12]. These cells can recruit other immune cells, be detected in lymphoid organs [13], present the antigen directly [14] or indirectly via dendritic cell cross-presentation [9]. Additionally, neutrophils were shown to reverse transmigrate and re-enter the circulation from the inflammation site through a mechanism that involved the activation of a pro-inflammatory phenotype [15,16,17].

In light of this new understanding of neutrophil biology, it is not surprising that their role in tumor progression has finally started to be evaluated.

## 2. The Dual Role of Neutrophils in Cancer

Only recently have neutrophils and their role in cancer received the attention they deserved. Their homeostatic alteration as part of tumor induced Sweet’s syndrome (acute febrile neutrophilic dermatosis) [18], in leukemia and in solid tumors was first reported in 1964 and 1971, respectively [19,20]. In the 1980s, different studies in solid cancers correlated PMN blood concentration with a worse prognosis, shorter survival, and higher rates of metastasis [21]. More recently, intratumor neutrophils were shown to be an independent prognostic factor for overall survival in metastatic and localized renal clear cell carcinoma [22,23], head and neck squamous cell carcinoma [24], hepatocellular carcinoma [25], melanoma [26], gastric adenocarcinoma [27], colorectal [28] and pancreatic cancer [29].

Interestingly, in non-small-cell lung carcinoma (NSCLC) intratumoral neutrophils fail to show any association with overall survival [30], whereas the CD66b^+^ neutrophil to CD8 lymphocytes ratio is predictive of worse outcome [31], suggesting that the interaction of neutrophils and T cells might be important for the overall prognosis. This possibility is further suggested by the work of Eruslanov et al. [32] showing that at the early stages of lung cancer tumor infiltrating CD66^+^MPO^+^CD11b^+^CD15^+^ neutrophils can indeed orchestrate the global anti-tumor T cell response. In contrast, at more advanced stages of disease, a distinct CD11b^+^CD14^−^CD15^+^CD33^+^ population of low-density, neutrophil like cells (also known as gMDSC) become detectable and is associated with T cell impairment and immune suppression [33]. Indeed, the blood neutrophil to lymphocyte ratio (NLR) is becoming an important prognostic factor and indicates poor clinical outcome in hepatocellular [34,35], renal [36], ovarian [37], colorectal [38], gastric [39,40], pancreatic [41] nasopharyngeal [42] and breast [43,44,45] cancer.

The simplistic view that neutrophils can only favor tumor progression is challenged by different clinical studies. For example, Caruso et al. [46] showed that a high intratumoral neutrophil infiltration is associated with a better survival in advanced gastric cancer. Interestingly, this study also proposed the existence of an important sexual dimorphism: while high infiltration of neutrophils was associated with a reduced mortality in women, this positive effect was not found in men. However, it is important to note that in this study, neutrophils were evaluated by H&E. Thus, considering neutrophils’ sexual dimorphism (i.e., presence of nuclear drum-stick in the female neutrophils), it is possible that PMN were simply better quantified in female patients. An alternative and more interesting hypothesis is that the functional sexual dimorphism (see below) that characterizes human neutrophils may play a role in their polarization and interaction with the tumor.

More recently, Sconocchia et al. [47] showed that the infiltration of CD33^+^HLA-DR^−^CD16^+^ neutrophils within the tumor is associated with better survival of patients with colorectal cancer. In a similar study double staining against neutrophil markers CD15 and myeloperoxidase (MPO) revealed that, while the number of tumor infiltrating MPO positive neutrophils was an independent favorable prognostic factor in patients with colorectal cancer, no significant association was found with the CD15 marker [48]. This suggests the presence of neutrophil subsets with different activities or functions within the tumor.

It is important to also remember that neutrophils, together with NK cells, play a key role in antibody-dependent cell-mediated cytotoxicity (ADCC) and have been shown to mediate the therapeutic effect of rituximab and trastuzumab in breast cancer [49] and lymphoma [50,51,52].

NETs are structures actively extruded by neutrophils in response to infection and are composed of DNA associated with antimicrobial peptides and proteins. These structures play an important anti-microbial role by trapping and killing invading pathogens. The role of NETs in tumor progression is still unclear, although human malignancies have been proposed to predispose circulating neutrophils to NETosis [53]. This can result in the trapping of circulating tumor cells that may facilitate neoplastic cell arrest and cancer associated thrombosis [54]. However, it is still difficult to evaluate the role of this process in tumor progression: although NETs can facilitate neoplastic cell arrest and subsequent invasion in the distal organs [55], it is important to remember that NETs are decorated with enzymes such as MPO, which are important for neutrophil mediated tumor cytotoxicity [56]. Additionally, NETs were proposed as a driving force by which neutrophils can transfer the antigen to professional APCs [9]. Thus, the overall effect of NETs in tumor progression might be complex and dependent on both the genetics of the neoplastic cells (i.e., resistance to oxidative stress), as well as the site where the NETosis occurs since efficient neoplastic seeding is dependent on the soil.

## 3. A gMDSC by Any Other Name Would Smell as Suppressive

Given the recently renewed interest of the role of neutrophils in cancer, a debate is growing in the scientific community on whether gMDSC (also called PMN-MDSCs) should just be called neutrophils, or if neutrophils with a suppressive and pro-tumoral phenotype should be called gMDSCs. Semantically, the name neutrophils is derived from the capacity of these cells to be colored equally by acid and basic dyes, whereas gMDSC describes a subset of myeloid cells expressing neutrophilic markers (i.e., mouse Ly6G or human CD15, CD66b) and characterized by a suppressive phenotype. MDSCs were known in the late 1970s as natural suppressor cells [57], and since the late 1990s have been rediscovered; their significant role in cancer and other diseases has become increasingly recognized. The biology of these cells are reviewed by Umansky group (German Cancer Research Center, Heidelberg, Germany). in this special issue. Thus, in this review we highlight the similarity and differences of these cells from neutrophils.

MDSCs have been shown to play a key role in tumor progression by regulating not only T [58,59,60] and NK [61,62] cell anti-tumor activity, but also by promoting tumor neo-vascularization [63], neoplastic cell invasion in the surrounding tissues [64] and the seeding of neoplastic cells in the distal site [64]. Additionally, in breast cancer, MDSCs have been suggested to form a pre-metastatic niche that prepares the soil for the invasion of neoplastic cells [65,66]. Finally, MDSCs have been implicated in endothelial to mesenchymal transition (EMT) [64] promoting neoplastic cell stemness [67].

Similarly, neutrophils have been related with a multitude of different mechanisms involved in tumor progression. They can directly sustain tumor growth by, for example, favoring tumor angiogenesis [68,69] and producing chemokines and growth factors able to modulate the tumor cell proliferation [69]. They can also induce immune suppression [33,70,71] and favor extracellular matrix degradation, neoplastic EMT, and generation of the pre-metastatic niche thus facilitating cancer cell spreading to distal site [69,72,73,74,75,76,77].

Thus, gMDSCs share many biological and clinical features associated with the subset of neutrophils with a pro-tumoral phenotype.

The absence of consolidated phenotypic markers able to differentiate gMDSC from neutrophils complicates the comparison of these two cellular subsets within the same host although physical properties (i.e., density) may be used to differentiate the suppressive population from the large majority of “normal” neutrophils.

To overcome the phenotypic limitations Youn et al. [78] compared Ly6G^+^ neutrophils from naïve mice with Ly6G^+^gMDSCs isolated from tumor bearing mice. This analysis reveals important phenotypic and functional differences (Table 1). Both cell types exhibit a PMN like phenotype, although later studies from Sagiv et al. [79] suggest that while neutrophils are more homogenous and uniformly characterized by the classic segmented nuclei, gMDSC are more heterogeneous and comprise immature subsets with ring-shaped nuclei as well as “mature” elements with segmented nuclei. At the moment it cannot be excluded that this heterogeneity reflects a convergent differentiation of different hematopoietic cells toward a similar functional and phenotypic state. Indeed, gMDSC were shown to differentiate from both monocytic MDSC [6] (Figure 1) as well as “classical” high density neutrophils [79].

In flow cytometry analysis, compared to non-suppressive neutrophils, gMDSC are characterized by an higher forward scatter (FSC) and lower expression of CD11b molecules [79]. These characteristics are reflected by the lower density of gMDSC that, both in mice and humans, segregate in ficoll gradient with the mononuclear cells rather than with the PMN [6,79]. Beside the slightly lower CD11b expression in gMDSC, in mice, most of the classical markers (i.e., Ly6G, Ly6C, IL4Rα, S100A8, S100A9, CCR5 and CXCR4) used to define neutrophils or gMDSCs are not differentially expressed [6]. However, contrary to the neutrophils, gMDSCs were reported to express CD115 as well as the CD244 marker that is normally expressed by NK cells and immature elements of the hematopoietic lineage [6]. Intracellular markers, such as LAMP2 and retinoblastoma 1, expressed by classical neutrophils but absent in gMDSCs can be useful to discriminate between the two populations [6]. Additionally, compared to “classical” neutrophils, gMDSC seem to be characterized by a slightly higher expression of arginase 1 [6], myeloperoxidase [6], and 12–15 lipoxygenase [79] activity and production of reactive oxygen species. Compared to gMDSCs, neutrophils have much higher phagocytic activity as well as a higher production of IFN-γ and TNF-α [6].

In humans, two similar subsets of neutrophil-like cells can be identified [79]: the high density FSC-A^low^CD11b^high^ neutrophils, consistent with classical neutrophils, are anti-tumoral, while the low density FSC-A^high^CD11b^int^ neutrophils, phenotypically similar to gMDSCs, are characterized by a strong immunosuppressive and pro-tumoral activity [79].

In summary, although differences can be found between neutrophils, gMDSCs and non-suppressive neutrophils, it is still unclear whether gMDSC are just a subset of neutrophils, neutrophils with a different activation or polarization state, or terminally differentiated cells with a neutrophil-like phenotype. Few studies have tried to answer this question experimentally, however, a definitive consensus is still not reached and in a majority of the cases, functionally similar cells are called either pro-tumoral neutrophils (also called N2 neutrophils) or gMDSCs. Additional studies to determine the origin of these cell subsets are needed to clearly address this important question. Considering the intrinsic limitation of a name, and that a consensus has not been reached, in this review, the terms of gMDSC and pro-tumoral neutrophils will be used as synonyms.

## 4. Sexual Dimorphism, Neutrophils and gMDSCs

One of the less studied aspects of PMN biology in cancer is the sexual dimorphism of granulocytes. Since 1954 [80] it has been known that the nucleus of granulocytes from women is characterized by the presence of a drumstick structure; defined by a 1.5 µm diameter head attached to the body of the nucleus by a thin filament. Although it is unknown whether this structure, derived by the inactivation of the extra X-chromosome [81], has direct functional consequences, analysis of neutrophils isolated from men and women reveals important differences as a consequence of the different exposure of hematopoietic cells to sex hormones in males and females.

Neutrophils are the first immune cells to reach the site of injury and infection. Interestingly, in a rat model of traumatic hemorrhagic shock or thermal injury, early neutrophil accumulation and respiratory burst at the site of injury was markedly increased in the male compared to the female in the proestrus period [82]. Castration experiments showed that neutrophil accumulation in the male rat was reduced, suggesting a role for androgens in the process [82]. Similarly, experiments activating neutrophils from females during the metestrus, diestrus, and proestrus period show estrus cycle dependent variation with the maximal resistance to activation during the proestrus. This suggests that estrogen may impair neutrophil activation. Neutrophils express estrogen receptors (ERα and ERβ) and thus, it is not surprising that they might be differentially regulated by the different concentrations in men and post-menopausal women (<40 pM) and fertile women (variable up to 4–500 pM during ovulation). Interestingly, nitric oxide synthase, an enzyme implicated in neutrophils and MDSCs immunosuppression, is increased in neutrophils during ovulation in human or after incubation with the 17-beta-estradiol estrogen [83]. The possible involvement of estrogen in neutrophil biology is also suggested by the attenuated morbidity of influenza A virus infected ovarectomized mice treated with 17-beta-estradiol [84]. In this model, neutrophils were slightly reduced in the lung at the early time point (1–5 days after infection) in 17-beta-estradiol treated mice, whereas at Day 7 CD11b^+^Ly6g^+^ neutrophils were markedly increased. Importantly, these changes were associated with a decrease of CCL2 and an increase of CCL3 and CXCL1 [84]. Depletion of Ly6G^+^ neutrophils in estrogen treated mice did not alter virus titer and replication in the host but normalized chemokine concentration and decreased overall virus related morbidity [84], suggesting that the excessive activation of neutrophils may mediate the virus induced toxicity. Additionally, this study suggested that 17-beta-estradiol can regulate the polarization or activation of recruited neutrophils.

In a model of Coxsackievirus group B type 3 (CVB3) induced myocarditis, female BALB/c mice showed a reduced morbidity (reduced body weight loss, serum CK activity, and myocardial inflammatory infiltration) and mortality compared to age matched male mice [85]. Immunophenotyping reveals a marked increase of post-infection mMDSC infiltrating the heart of the female whereas, in male, myocardial infiltration was mostly characterized by Ly6G^+^ cells. Furthermore, mMDSCs from female mice were more suppressive and stronger inducers of regulatory Treg than their male counterpart. Importantly, while adoptive cell transfer of MDSCs from females significantly reduced myocarditis in male recipients, no effects were detected when male donors were used [85]. Another study [86] propose that estradiol dependent induction of MDSC-like cells plays an important role in the sexually dimorphic etiology of systemic lupus erythematosus, a multi-systemic autoimmune disease that develops at a female-to-male ratio of 9:1 [86]. In patients with SLE a higher percentage of immature MDSC like cells are present in the blood of women compared to men of comparable age. Experiments in mice seem to suggest a role for Estradiol and TNF-α in MDSC accumulation. However, the extremely low variation seen in patients and in the preclinical data question the validity of the study which has not yet reproduced by other groups [86]. Interestingly, a preclinical study [87] using naïve mice showed a sexually dimorphic immune modulation of PDE5 inhibitors, a class of drugs currently tested to downregulate MDSC action in cancer patients [88,89]: in naïve mice PDE5 seemed to reduce NK and memory T cells in male, while increasing these subsets in female. Additionally, PDE5 reduced the CD11b^+^GR1^+^MDSCs in female but not in male mice [87]. In tumor bearing mice [90] and in HNSCC patients undergoing PDE5 blockade, we did not observe this sexually functional dimorphism, and a reduction of MDSCs in the blood and in the tumor was found after treatment in both men and women. However, it is important to note that the median age of the women in our clinical trial was 60 (range 40–76) and thus, the large majority were unlikely to be subject to estrus hormones fluctuation.

Early experiments [91] using calves treated with high doses of progesterone showed a higher mobility of neutrophils from treated calves compared to control animals. Progesterone was suggested to modulate β_2_ adrenergic receptor which plays a sexually dimorphic role in PMN biology. Chemotaxis to β_2_ adrenergic receptor agonist is higher in women’s neutrophils even though there is no apparent difference in β_2_ adrenergic receptor expression between the sexes [92]. Experiments with β_2_ adrenergic receptor KO mice revealed that females exhibit an higher recruitment of neutrophils in response to LPS that could be recovered in male by the genetic depletion of β_2_ adrenergic receptor [92].

Testosterone is a major circulating androgen, which is synthesized by testicular Leydig’s cells and in small amounts by the adrenal glands. The effect of this hormone on neutrophils is less studied, however, physiological level of testosterone seems to modulate neutrophils’ function [93]. Specifically, the lower range of the physiological concentration of testosterone (10 nM) was sufficient to drastically lower superoxide production in neutrophils and increase their phagocytic activity and glutathione reductase activity [93].

Sex hormones may not only influence neutrophil chemotaxis and function, but also regulate their apoptosis [94]. Neutrophils spontaneously undergo apoptosis in vitro and in vivo. Evaluation of apoptosis in neutrophils harvested from fertile women or age matched men surprisingly revealed that men’s neutrophils are more prone to apoptosis than the women’s counterpart [94]. Interestingly, progesterone and/or estradiol added to the culture further reduced the apoptosis of neutrophils isolated from both sexes [94] by a mechanism that requires caspase 3 and 9 downregulation.

In summary, estrogen seem to reduce neutrophil mobility and inflammatory activity, whereas progesterone might increase chemotaxis. Both hormones seem to have an anti-apoptotic effect on neutrophils. Testosterone might reduce superoxide production and anti-microbial action while favoring neutrophils phagocytic activity. However, the role of sexual hormones in neutrophil biology and polarization is still an understudied area of research that may explain the contribution of PMNs in the gender preferential appearance and response to therapy of many human diseases.

## 5. Therapeutic Modulation of Neutrophils and gMDSC in Cancer

### 5.1. Improving Neutrophil Antitumor Activity

As mentioned above, neutrophils can exert an important anti-neoplastic activity directly and indirectly by releasing many chemokines, cytokines and growth factors able to orchestrate anti-tumor immunity. For example, forced expression of FAS-ligand in tumor cells promotes a rapid regression of experimental lymphoma, melanoma and hepatoma in immunocompetent and T cell deficient mice [95]. This T cell independent regression was shown to be exclusively dependent on FAS^+^ neutrophils [95,96]. However, later studies revealed that neutrophils were also able to promote long term tumor immunity [97] and protect mice from re-challenge via recruitment of DC and B cells and generation of anti-tumor antibodies [97].

These experimental mechanisms also seem to mediate the antitumor activity of the protein kinase C (PKC) modulator PEP005 (ingenol-3-angelate). This drug can drastically reduce tumor relapse by promoting the direct elimination of skin tumors after topical treatment. This effect seems to be mediated by neutrophils since their inhibition resulted in reduced inflammation and significant increase in tumor relapse rates following PEP005 treatment [98]. PEP005 favors neutrophil recruitment and activation via MIP-2/IL-8, TNF and IL-1 and tumor elimination via neutrophil mediated ADCC [98,99,100].

Although natural killer cells have been considered as main effector cells in ADCC, a role for the innate mononuclear phagocytes and neutrophils has been shown as well [101]. To date, the mechanism involving neutrophils in ADCC is not completely understood, but evidence indicates that neutrophil cytotoxic ability is greatly enhanced in the presence of target-specific mAbs [102]. Neutrophils constitutively express the low to intermediate affinity IgG Fc receptors FcγRIIIb (CD16) and FcγRII (CD32), whereas the high affinity Fc receptor for IgG, FcγRI or CD64, is upregulated in the presence of IFN-γ or G-CSF [103]. Moreover GM-CSF enhances the anti-tumor neutrophil-mediated ADCC through increased expression of CD11/CD18 molecules [104].

FcαRI (CD89) is considered the master mediator of neutrophil’s ADCC for many targets including HER-2, EGFR [105], HLA class II and CD20 [106,107], CD30 , and CEA [108].

FcγRIIa stimulation leads to potent neutrophil-mediated and antibody-dependent killing of tumor cells [109]. FcγRIIIb is almost exclusively on neutrophils [110] and basophils [111]. Because of its low affinity to the antibody, this receptor fails to induce efficient ADCC unless ad hoc modifications are performed on the fc region of the therapeutic antibody. For example, this was successfully accomplished by removing fucose group on ritubimax that drastically enhanced anti-CD20-mediated apoptosis in tumor cells [112] by increase antibody affinity for FcγRIIIb [113].

Given the importance of anti-cancer antibodies in the treatment of human malignancies, modification of their structure aimed at improving their affinity for neutrophil’s FcγRIIIb and promoting the accumulation of “N1”neutrophil at the tumor site are promising strategies to increase the efficacy of this class of therapeutics.

### 5.2. Targeting Key Factors Responsible for Neutrophils Pro-Tumoral Polarization

Considering that in advanced stage of tumor development neutrophils are generally polarized toward a pro-tumoral “N2” phenotype, that gMDSC are accumulated in the tumor bed and systemically, and that neutrophils and gMDSCs are constantly regenerated, therapeutic strategies aimed to impair the pro-tumoral polarization of neutrophils/myeloid cells are being investigated. One of the major factors determining neutrophil commitment toward a pro-tumor phenotype is TGFβ. TGFβ-1, -2 and -3, secreted as inactive homodimeric polypeptides, are the primary mediators of TGFβ signaling that mediate TGFβ receptor dimerization and/or the activation of the canonical SMAD pathways as well as, among others, the non-canonical PI3K-AKT, RHOA and MAPK pathways [114]. In lung adenocarcinoma and mesothelioma models, TGFβ induces neutrophil polarization toward a pro-tumoral phenotype, characterized by ARG1 expression and low levels of TNF, CCL3 [115]. Blockade of TGFBR1 signaling via the kinase inhibitor SM16, induces the accumulation at the tumor site of hypersegmented neutrophils able to directly kill tumor cells and mediate a CD8^+^ cell dependent immunity by the secretion of pro-inflammatory cytokines [115]. Granulocyte depletion significantly reduces TGF-β blockade anti-tumor efficacy [115].

Type 1 Interferons (IFN) and in particular IFN-β seem to play the opposite role of TGF-β in neutrophil polarization favoring an anti-tumoral “N1” phenotype. Indeed, IFN-β deficient mice develop fast growing and highly angiogenic tumors characterized by significant infiltration of neutrophils expressing high levels of c-myc and STAT3 [68]. In vitro treatment of these tumor infiltrating neutrophils with low INF-β doses downregulates STAT3 and cMYC expression and favors a “N1” re-polarization [68]. Similarly, in the absence of IFN-β, the primary lesion and pre-metastatic lung of mice bearing mammary carcinomas are characterized by the presence of neutrophils with ring-shaped nuclei, a prolonged life span, poor tumor cytotoxicity, low NETs expression and low expression of ICAM1 and TNF-α molecules [116]. IFN-β treatment in mice altered TAN polarization towards anti-tumor N1 [116].

Since Type I IFN (IFN-α) therapy is currently approved for high risk melanoma patients, Andzinski et al. compared neutrophils from melanoma patients undergoing IFN-α therapy with those isolated from untreated patients [116]. This analysis revealed that neutrophils from IFN-α treated patients show significantly lower expression of CXCR2 and a significant increase in frequency of PMN with band-shaped nuclei [116]. Although the authors suggest that these data confirm the mouse finding, a lower CXCR2 and an immature phenotype is often associated with pro-tumoral low density neutrophils [115].

Given the importance of IFN therapy in human malignancy, it is extremely important to evaluate functionally the effect of this therapy as well as differences between IFN-α and β on neutrophil polarization.

With the approval of 3 HDAC inhibitors for cancer therapy by the US Food and Drug Administration (FDA), epigenetic modification of neutrophils to modulate their polarization has captured interest. Particularly important in this field is the work of Youn et al. [6] showing that HDAC2 dependent epigenetic silencing of RB1 in monocytic MDSC is necessary for the generation of gMDSC. Importantly, HDAC inhibition restores RB1 expression and promotes the differentiation of mMDSC towards macrophages and DCs [6].

Although neutrophils from healthy donors express RB (Table 1) [6], it is still unknown whether HDAC inhibition and RB1 expression is sufficient to induce the differentiation of tumoricidal neutrophils.

### 5.3. Targeting Neutrophils Expansion and Recruitment to the Tumor Site

Given the fact that most neutrophils in advanced stage tumors are pro-tumoral, strategies are being tested to limit PMN trafficking at the tumor site. The redundancy of chemotactic signals can raise concerns on the efficacy of these strategies however, interesting results were obtained with these approaches. In patients with hepatocellular carcinoma the presence of high expression of CXCR2 and its ligands CXCL5 and CXCL8 (IL-8) on tumor cells are associated with neutrophil recruitment and neoplastic cell spreading [117].

The administration of antibodies against IL-8 (ABX-IL-8) attenuated the growth of bladder cancer xenograft models [118], and decreased the tumorigenic and metastatic potential of A375SM and TXM-13 melanoma xenograft models [119]. Moreover, reduced tumor growth and increased response to docetaxel was induced in ovarian tumor xenografts after IL-8 suppression via liposome-encapsulated small-interfering RNA (siRNA) [120]. Numerous chemotherapy agents (e.g., 5-fluorouracil, Adriamycin, dacarbazine, paclitaxel) were shown to induce IL-8 expression and secretion in cancer cells [121,122,123] as a mechanism of resistance; targeting IL-8 signaling may increase the sensitivity of cancer cells to conventional chemotherapy and novel treatment strategies.

As mentioned above, chemotactic factors are usually redundant. Indeed, other ligands such as CXCL1, CXCL2, CXCL3, CXCL5, CXCL6, CXCL7, oxysterol, and migration inhibitory factor (MIF) are able to induce neutrophil recruitment and activation via CXCR1 and CXCR2 [124,125,126].

Targeting receptors instead of ligands offers the advantage of making it easier to overcome the overproduction of chemokines secreted by tumors and resolve some of the chemokine redundancy. Thus, strategies have been developed to inhibit CXCR2 and have shown promising results [127]. In the CCSPcre/K-rasG12D K-ras mutant mouse model of lung cancer the selective inhibition of CXCR2 led to a reduction in neutrophil infiltration in the lungs and diminished tumor growth [128]. Similarly, in a model of invasive intestinal adenocarcinoma (*AhCreER*; *Apc^fl/+^*; *Pten^fl/fl^* mice), CXCR2 deficiency was shown to suppress inflammation-driven tumorigenesis in the skin and intestine, as well as spontaneous adenocarcinoma formation [129]. In AB1 and LCC mouse models the administration of CXCR2 antagonist SB225002 reduced tumor growth likely by limiting neutrophil recruitment and their role in immunosuppression and angiogenesis. Similar results were obtained by inactivating the CXCR2 ligand oxysterols with SULT2B1b [124]. However, it is important emphasize that CXCR2 can also be expressed in stromal cells and by tumor cells. In a mouse model of pancreatic ductal adenocarcinoma, it was demonstrated that tumor progression could be suppressed using a CXCR2 inhibitor via dysregulation of stroma-tumor signaling [130].

CXCR6 and its ligand CXCL6 are involved in neutrophil recruitment in both a direct and indirect manner. Tumor cells and lymphocytes express CXCR6 on their surface and evidence of CXCR6 expression in tumor infiltrating neutrophils was found in patients with pancreatic carcinoma [131]. CXCR6 expression in the tumor is associated with high neutrophil infiltration and poor prognosis in HCC patients. Stimulation of tumor cells via CXCR6 induces the production of CXCL8 which induces the recruitment of neutrophils. Moreover, CXCL16, a chemoattractant for CXCR6 expressing tumor cells, has been linked to an increase in tumor cell migration and invasion in prostate cancer [132] and pancreatic ductal adenocarcinoma (PDAC) [133]. CXCR6 stimulates the conversion of mesenchymal stem cells into cancer-associated fibroblasts, facilitating tumor metastasis [134], whereas CXCL16 promotes tumor proliferation and migration [135]. Thus, a therapeutic approach able to target the CXCR6-CXCL16 axis could have a dual role in tumor therapy, leading to diminished infiltration of neutrophils and a reduced invasiveness of cancer cells.

It is important to note that even when chemokine receptors are targeted, the redundancy and pleiotropism of these pathways may dramatically reduce therapeutic efficacy. This possibility is exemplified by the disappointing results of clinical trials using CCR1 antagonists for the treatment of rheumatoid arthritis, multiple sclerosis, and COPD [136]. Despite the fact that CCR1 is upregulated in all of these diseases and that inhibition of CCR1 or its ligands demonstrated beneficial effects in animal models, clinical data showed lack of efficacy. These disappointing results may be explained by the presence of other receptors with similar function [136] that can compensate CCR1 inhibition. Similar disappointing results contradicting animal model data were obtained when CCR2 [137] or CCR5 [138] antagonists were tested in patients with rheumatoid arthritis. In cancer, these antagonists have just started clinical experimentation, thus, it is still early to evaluate their efficacy. In patients with bone metastases, the humanized anti-CCR2 antibody MLN1202 was able to reduce urine *n*-telopeptide (a bone turnover rates marker) in 14% of the patients suggesting at least some therapeutic efficacy. A phase 1 trial for the CCR5 antagonist Maraviroc in colorectal liver metastasis is currently being performed, but no results are yet posted (NCT01736813).

### 5.4. Targeting Neutrophils Relevant Molecules for Tumor Growth and Metastasis Formation

Neutrophil elastase (NE), a serine proteinase characterized by broad substrate specificity, is particularly important in neutrophil function and can mediate the cleavage of nearly all components of the extracellular matrix including cytokines, cytokine receptors, integrins, and inert elastic fibers [139]. Considering the importance of the extracellular matrix in tumors, NE may play a key role in tumor invasion into the surrounding tissues. For example, NE mediated cleavage of the tumor cell adhesion molecule E-cadherin may favor tumor spreading and metastasis by allowing tumor dissemination and favoring EMT [76,140]. Insulin receptor substrate-1 (IRS-1), the binding partner of the p85 regulatory subunit of phosphoinositide 3-kinase (PI3K), is another target of NE activity. IRS-1 degradation increases p85 bioavailability and, via interaction with platelet derived growth factor receptor (PDGFR) and other factors, significantly increases neoplastic cell proliferation [141].

In accordance with these NE pro-tumoral roles, the usage of the specific NE inhibitor in the treatment of pancreatic cancer reduced tumor proliferation and migratory capacity [142]. Similar results were obtained by the use of Elafin, a natural endogenous elastase inhibitor [143]. Elafin has also been shown to induce apoptosis by inhibiting elastase-mediated cleavage of CD14 [144]. Moreover, part of the antitumor activity of curcumin has been linked to the ability to block neutrophil elastase-induced tumor proliferation via upregulating α1-antitrypsin expression in lung cancer in vitro and in vivo [145].

Since inhibition of neutrophil elastase is considered an important step in the treatment of pulmonary diseases like chronic obstructive pulmonary disease (COPD) and in alleviating the symptoms of cystic fibrosis; many inhibitors have been designed and are currently being tested in clinical trials [146] which target elastase as part of cancer treatment. However, since neutrophil elastase plays a pivotal role in the innate immune response (bacterial infections), in tissue remodeling processes, and in the onset and resolution of inflammation, the inhibition of elastase in the clinic raises some safety concerns related to possible transient immunosuppression and interference with proper wound healing.

Metalloproteinases (MMPs) are involved in many steps of cancer progression. Their presence and relative abundance is often related to cancer stage and patient prognosis [147]. Tumor-associated neutrophils (TANs) are important for pro-matrix metalloproteinase-9 (pro-MMP9) release at the tumor site, especially at early stages of tumor development. The presence of pro-MMP9 expressing TANs has been related to the occurrence of high levels of metastasis both in experimental models [72,148] and in cancer patients [25]. In particular, the presence of lumen-containing blood vessels seems to be directly related to the MMP9 activity suggesting that pro-MMP9 directly affects the microarchitecture of the newly formed vasculature. Experiments performed with WT of MMP9-KO mice demonstrated that the tumor vasculature developing in MMP9-KO was represented mainly by collapsed capillaries [148].

Pro-MMP9 facilitates VEGF bioavailability [149], and its interaction with VEGFR. In particular MMP-9 cleavage of the connective tissue growth factor (CTGF) in the VEGF_165_-CTGF complex restores the angiogenic function of VEGF_165_ isoform, that is inhibited in the complexed form. [149,150]. Moreover MMP-9 directly promotes cell migration by altering cell-cell adhesion function [151] facilitates tumor angiogenesis and MDSCs/neutrophil recruitment by regulating the bio-availability of VEGF and its interaction with VEGFR [149,150].

Inhibition of MMP9 by the endogenous inhibitor TIMP-1 reduces angiogenesis in chorioallantoic membrane assays [152] and promotes cell-cell adhesion of neoplastic cells [151]. These results may be relevant in vivo since macrophage polarization toward a “M2” phenotype is characterized by a significant decrease of TIMP-1. Conversely, forced “M1” polarization is characterized by TIMP-1 induction and a reduction of MMP9 mediated angiogenesis [148]. Interestingly, the ability of aggressive tumor cells to intravasate is linked with their ability to recruit inflammatory neutrophils delivering their unique TIMP-1-free proMMP-9 [72].

The selective interference with prometastatic neutrophil functions represents an attractive new strategy for cancer treatment. A recent study has demonstrated that in the MMTV-PyMT breast cancer model, neutrophils express high levels of the lipids leukotriene B4 (LTB4) and cysteinyl leukotrienes C4, D4 and E4 (LTC/D/E4), products of the arachidonate 5-lipoxygenase (Alox5) enzyme [153]. These molecules seem to specifically increase the proliferation of metastasis-initiating cells in MAPK/ERK kinases (MEK)1- and 2-mediated, pERK1/2-dependent manner indicating that LTs provide a selective proliferative advantage to cancer cells with intrinsically higher tumorigenicity [154]. In fact, administration of zileuton, an inhibitor of Alox5 used for the treatment of asthma, was shown to inhibit the formation of lung metastasies [154,155]. Lung metastases can also be inhibited by targeting neutrophil proteases as elastase and cathepsin G. These proteases specifically target thrombospondin-1a (Tsp-1) a potent anti-tumorigenic factor, resulting in its degradation [156].

## 6. Conclusions

Neutrophils are finally being recognized for the important role they play in tumor progression. Depending on their polarization, these cells can promote tumor growth by favoring tumor angiogenesis, metastasis, and immune suppression; or they can promote tumor eradication by a direct tumor cytotoxicity and by orchestrating the adaptive immune response against the tumor.

Still, the differentiation and/or polarization pathways, the role and cooperation of different tumor derived factors on neutrophil chemotaxis and polarization, the influence of sexual hormones on the biology of these cells, and the mechanisms that regulate their pro-tumoral and anti-tumoral action needs to be defined. Additionally, there is the critical need for the identification of functional markers able to discriminate between the different PMN subsets and polarization states. Nevertheless, promising therapeutic strategies aimed at eliminating or inhibiting pro-tumorigenic neutrophils, while increasing the frequency and or activity of anti-tumoral neutrophils, are being developed.

In summary, in the 100 years since Metchnikoff’s death, many studies are still required to dissect the biology of neutrophils that may lead to important therapeutic applications for the immunological treatment of tumors.

## Figures and Tables

**Table 1:** Phenotype of gMDSCs and Neutrophils.

Variable group	Variable	gMDSC	Neutrophils	References
Physical characteristics	Morphology	PMN heterogeneous: band, segmented and ring shaped nuclei (mature and immature)	PMN homogeneous segmented (mature)	[77]
	Density	Low	High	[78]
	FSC	High	Low	[78]
Surface markers	CD11b	+++	++++	[77,78]
	Ly6G	++++	++++	[77]
	Ly6C	+++	+++	[77]
	IL4Ra	+	+	[77]
	S100A8	+	+	[77]
	S100A9	+	+	[77]
	CCR5	+	+	[77]
	CXCR4	+	+	[77]
	CD115	+	-	[77]
	CD244	++	-	[77]
Intracellular markers	LAMP2	+	+++	[77]
	RB1	-	+++	[6]
Enzymes	MPO	++	+	[77]
	Arginase	+++++	+++	[77]
	12/15-lipoxygenase	++	+	[78]
Others	Phagocytosis	+	++	[77]
	TNFα	+	++++	[77]
	IFNγ	+	++++	[77]
	ROS	++	+	[77]

## Abbreviations

The following abbreviations are used in this manuscript:

PMN	polymorphonuclear cells
GMP	granulocytes-monocytes progenitors
MDSCs	myeloid derived suppressor cells
RB1	retinoblastoma 1
NETs	neutrophils extracellular traps
HDN	high density neutrophils
ROS/RNS	reactive oxygen species/reactive nitrogen species
TNFα	tumor necrosis factor alpha
TRAIL	TNF-related apoptosis-inducing ligand
ADCC	antibody-dependent cell-mediated cytotoxicity
TDFs	tumor derived factors
EMT	endothelial to mesenchymal transition
NSCLC	non-small-cell lung carcinoma
MPO	myeloperoxidase
gMDSC	granulocytic-myeloid derived suppressor cells
NLR	neutrophil to lymphocyte ratios
H&E	hematossilin and eosin staining
HLA-DR	human leukocyte antigen—antigen D Related
NKs	natural killer cells
APCs	antigen presenting cells
LAMP2	lysosome-associated membrane protein 2
IFNγ	interferon gamma
ER	estrogen receptor
CVB3	coxsackievirus group B type 3
CK	creatine kinase
mMDSC	monocytic-myeloid derived suppressor cells
Treg	T regulatory
SLE	systemic lupus erythematosus
PDE5	phosphodiesterase type 5 inhibitor
HNSCC	head and neck squamous cell carcinoma
LPS	lipopolysaccaride
DCs	dendritic cells
PKC	protein kinase C
MIP-2	macrophage inflammatory protein 2
IL-8	interleukin-8
IL-1	interleukin-1
mAbs	monoclonal antibodies
GM-CSF	granulocyte-macrophage colony-stimulating factor
CEA	carcinoembryonic antigen
HER-2	human epidermal growth factor receptor 2
EGFR	epidermal growth factor receptor
TGFβ	transforming growth factor beta
SMAD	Contraction of Sma and Mad (Mothers against decapentaplegic)
PI3K	phosphoinositide 3-kinase
RHOA	Ras homolog gene family, member A
MAPK	Mitogen-activated protein kinases
ARG1	Arginase 1
TGFBR1	transforming growth factor beta receptor 1
STAT3	Signal transducer and activator of transcription 3
ICAM1	Intercellular Adhesion Molecule 1
TAN	Tumor Associated Neutrophil
3 HDAC	Histone deacetylase 3
FDA	Food and Drug Administration
HDAC2	Histone deacetylase 3
GSK-3β	Glycogen synthase kinase 3 beta
PDAC	pancreatic ductal adenocarcinoma
mTOR	Mammalian target of rapamycin
COPD	Chronic obstructive pulmonary disease
NE	neutrophil elastase
IRS-1	Insulin receptor substrate-1
PDGFR	platelet derived growth factor receptor
MMPs	Metalloproteinases
VEGF	Vascular endothelial growth factor
VEGFR	Vascular endothelial growth factor receptor
TIMP-1	metallopeptidase inhibitor 1
MMTV-PyMT	mouse mammary tumor virus polyoma virus middle T antigen
LTB4	lipids leukotriene B4
Alox5	5-lipoxygenase
Tsp-1	thrombospondin-1a
